# Stable Near-Infrared Photoluminescence of Single-Walled Carbon Nanotubes Dispersed Using a Coconut-Based Natural Detergent

**DOI:** 10.1021/acsomega.1c04615

**Published:** 2021-11-02

**Authors:** Kota Hirayama, Masaki Kitamura, Ryo Hamano, Kazuo Umemura

**Affiliations:** Department of Physics, Tokyo University of Science, 1-3 Kagurazaka, Shinjuku 1628601, Japan

## Abstract

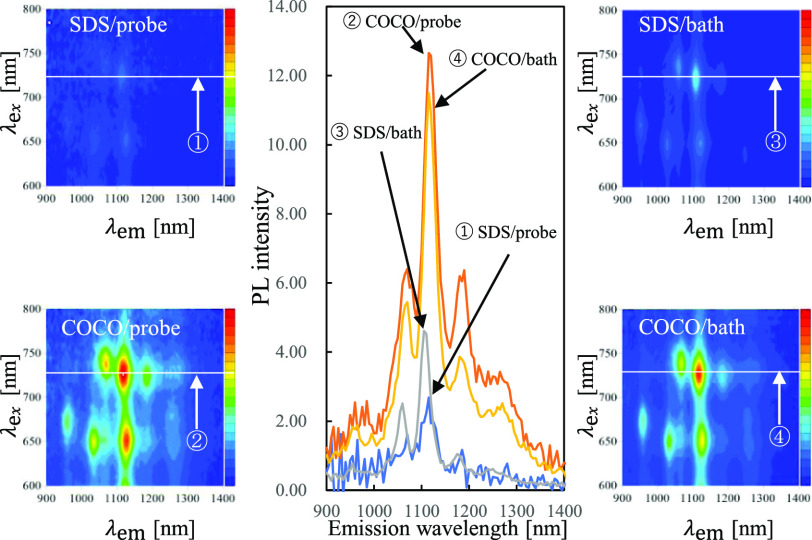

We prepared single-walled carbon nanotube (SWNT) suspensions in phosphate buffer solutions containing 1% of a coconut-based natural detergent (COCO) or 1% of sodium dodecyl sulfate (SDS). The suspensions exhibited strong photoluminescence (PL) in the near-infrared region, suggesting that the SWNTs, such as those with (9, 4) and (7, 6) chiralities, were monodispersed. Upon diluting the suspensions with a detergent-free phosphate buffer solution, the PL intensity of the SDS-containing SWNT suspension was significantly lower than that of the COCO-containing SWNT suspension. The COCO-containing SWNT suspension was more stable than the SDS-containing SWNT suspension. The SWNT concentration of the suspensions prepared via bath-type sonication was lower than that of the suspensions prepared via probe-type sonication. However, near-infrared (NIR) PL intensity of the SWNT suspensions prepared via bath-type sonication was much higher than that of the SWNT suspensions prepared via probe-type sonication regardless of the detergent. This suggested that the fraction of monodispersed SWNTs of the suspensions prepared via bath-type sonication was larger than that of the suspensions prepared via probe-type sonication, although the SWNT concentration was low. Our results indicated that COCO favored the fabrication of SWNT suspensions with stable and strong NIR PL, which are useful for various biological applications.

## 1 Introduction

The preparation of monodispersed carbon nanotube (CNT) suspensions is a basic and important step in developing CNT applications.^1−6^ Monodispersed single-walled CNTs (SWNTs) exhibit remarkable physicochemical properties, such as near-infrared (NIR) photoluminescence (PL).^7−11^ In particular, when SWNTs synthesized using the high-pressure carbon monoxide (HiPco) method are well monodispersed, a strong NIR PL can be observed upon irradiating the SWNT suspensions with visible light of a suitable wavelength.^12−15^ NIR PL is useful for various applications, such as optical switches and biosensors.^12,15−20^ For example, the detection of single mismatches in DNA sequences using monodispersed SWNTs has been reported.

Raw SWNTs are insoluble in water and can easily form bundles. However, the aforementioned physicochemical properties cannot be observed for bundled SWNTs.^21−24^ Several methods have been proposed for the preparation of monodispersed SWNT suspensions by encapsulating SWNT molecules. A typical method involves the use of synthesized surfactants, such as sodium dodecyl sulfate (SDS) and sodium cholate (SC).^25−30^ Appropriate amounts of SWNT powder are added to surfactant aqueous solutions, and the mixtures are sonicated using probe- rather than bath-type sonicators to separate the bundled SWNTs. Because surfactant molecules are adsorbed on isolated SWNT molecules and “wrap” the surface of SWNTs, the formation of SWNT bundles is prevented even after sonication is stopped. Wrapping methods using several organic molecules have been proposed. Single- or double-stranded DNA molecules are commonly used to wrap SWNTs utilized for biological applications.^31−35^ The affinity of DNA molecules for the surface of SWNTs is affected by the DNA sequence, and the DNA wrapping manner can be regulated by adjusting the DNA sequence.^36−38^ Synthesized polymers, such as carboxymethyl cellulose, have also been widely used for dispersing SWNTs.

Although dispersing SWNTs using SDS or SC is convenient and inexpensive, many researchers have demonstrated that dispersing SWNTs using surfactants results in unstable suspensions.^39−43^ Therefore, several researchers have proposed new methods for improving the stability of SWNT suspensions using surfactants.^44−49^

Recently, we prepared CNT dispersions using several coconut- and bamboo-derived natural detergents.^50^ We wrapped CNTs using eco-friendly green chemicals and subsequently prepared aqueous suspensions of SWNTs containing carboxylic groups (1.0–3.0 at. %) and multiwalled CNTs using a bath-type sonicator. However, we could not disperse bare SWNTs fabricated using the HiPco method using the bath-type sonicator. Furthermore, the fabricated SWNTs did not exhibit NIR PL because they were produced using an arc discharge method. SWNTs fabricated using the HiPco method include several chiralities, which exhibit strong NIR PL.^13,51,52^ Chirality, which is defined as the chirality vector (*n*, *m*), determines the structures and physicochemical properties of SWNTs. For example, the SWNTs with (9, 4) and (7, 6) chiralities, which are typically fabricated using the HiPco method, exhibit strong NIR PL.

In this study, we dispersed SWNTs fabricated using the HiPco method using a coconut-based natural detergent (COCO) for the first time. Probe- and bath-type sonicators were used to prepare SWNT suspensions. Unlike the SDS-wrapped SWNTs, the COCO-wrapped SWNTs exhibited stable NIR PL even at low COCO concentrations.

## 2 Results and Discussion

The typical method used to solubilize SWNTs with SDS is simple.^42^ Appropriate amounts of SWNT powder are added to an SDS aqueous solution, and the mixtures are sonicated. Probe-type sonicators have been widely used to achieve good results, although bath-type sonicators have also been used. SDS molecules attach to the surface of the debundled SWNTs during sonication; therefore, SWNTs can be dissolved in aqueous solutions. Thereafter, the samples are typically centrifuged to disperse the aggregates, and the supernatants are stored as “SDS-SWNT” hybrid suspensions.

**Figure 1:**
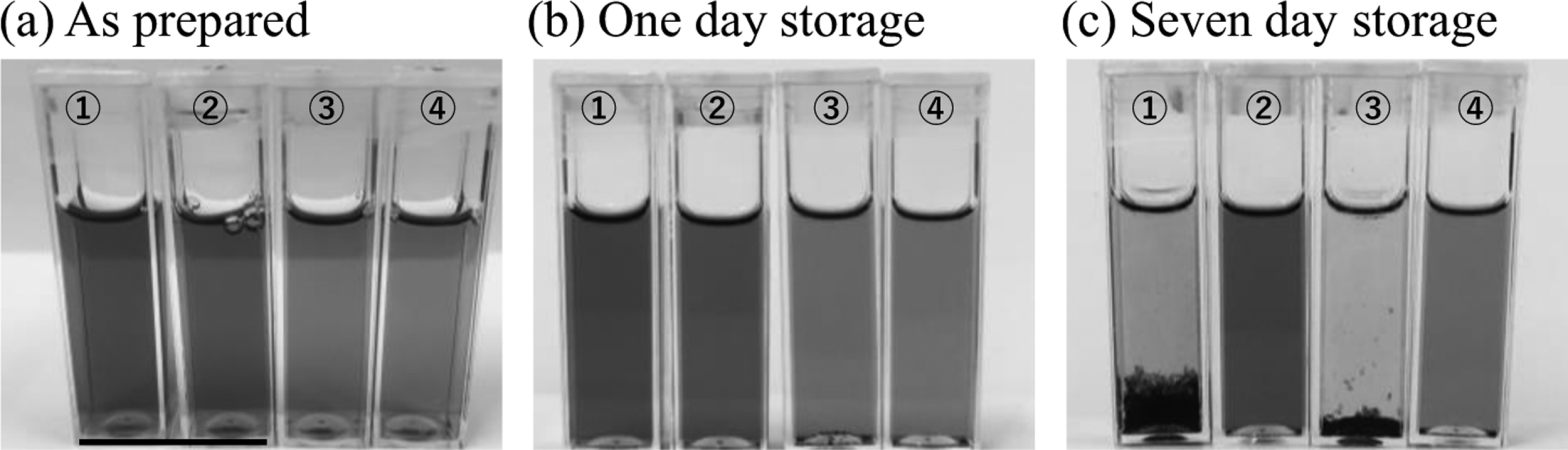
Photographs of single-walled CNT suspensions containing 1% of SDS (①,③) and the COCO (②,④) fabricated using probe- (①,②) and bath-type sonicators (③,④): (a) as-prepared, (b) after 1 day, and (c) after 7 days. The suspensions were diluted 10 times with a detergent-free phosphate buffer solution to a final detergent concentration of 0.1% and were subsequently stored.

We used a similar procedure to solubilize SWNTs utilizing the aforementioned COCO detergent. SWNT solubilization with SDS was performed in parallel for comparison. During our experiments, mixtures of the SWNT (final concentration of 0.5 mg/mL) and SDS or COCO detergent (final concentration of 1%) were sonicated using probe- or bath-type sonicators and the prepared suspensions are denoted as SDS/probe, SDS/bath, COCO/probe, and COCO/bath, respectively. The photographs of the prepared suspensions before centrifugation are illustrated in Figure 1. To evaluate the stability of the suspensions, each sample was diluted 10 times with a detergent-free phosphate buffer solution, such that the detergent concentration after dilution was 0.1%. No apparent aggregates or precipitates were observed in the samples immediately after dilution. After 1 day, the small amount of the precipitate formed in the SDS-containing SWNT suspension was sonicated using the bath-type sonicator. After 7 days, the SDS-containing SWNT suspensions contained abundant precipitates. These results suggested that the COCO-containing SWNT suspensions were more stable than the SDS-containing SWNT suspensions with a detergent concentration of 0.1%.

**Figure 2:**
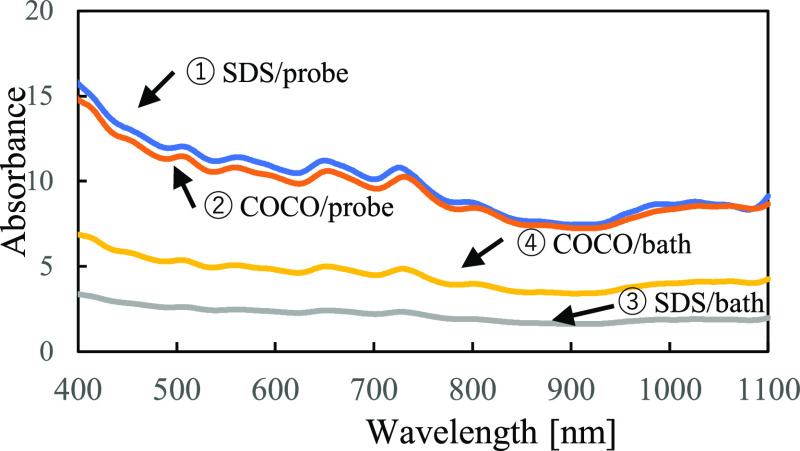
Absorbance spectra of single-walled CNT suspensions prepared using ① SDS via probe-type sonication, ② COCO via probe-type sonication, ③ SDS via bath-type sonication, and ④ COCO via bath-type sonication. For the absorbance measurements, suspensions were diluted 10 times with a detergent-free phosphate buffer solution until the detergent concentration was 0.1%, and the absorbance of each sample was measured immediately after dilution. Absorbance values of this figure were converted into absorbance of the original suspension (×10).

**Figure 3:**
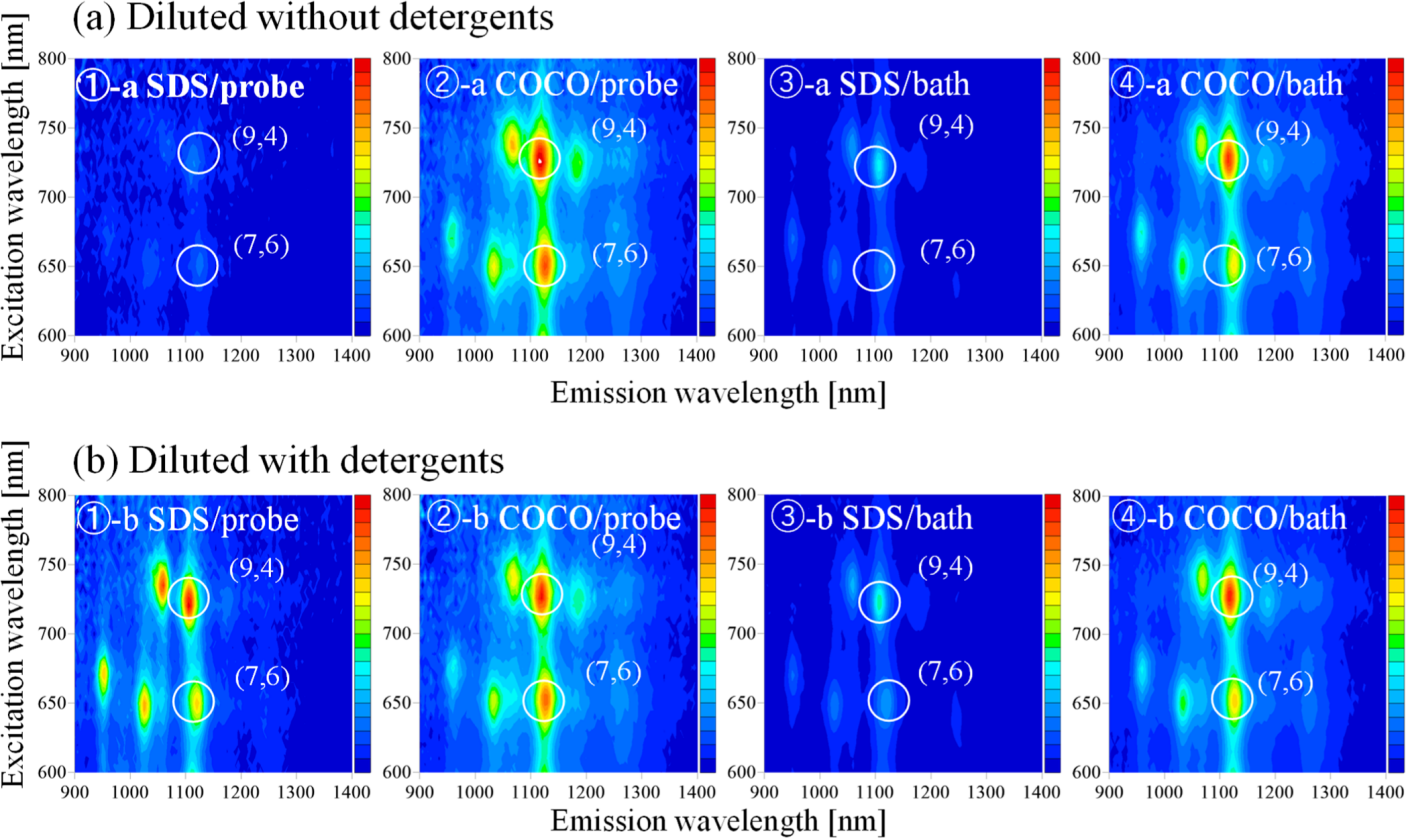
PL maps of single-walled CNT suspensions prepared using ① SDS via probe-type sonication, ②COCO via probe-type sonication, ③ SDS via bath-type sonication, and ④ COCO via bath-type sonication. The excitation and emission wavelength ranges were 600–800 and 900–1400 nm, respectively. Each suspension was diluted with (a) detergent-free or (b) detergent (1% SDS or 1% COCO)-containing phosphate buffer solution, such that absorbance of the suspension was 0.1. In the PL maps, PL intensity values were converted into those of the original suspensions for simple comparison. The raw data before conversion are shown in Figure S3.

**Figure 4:**
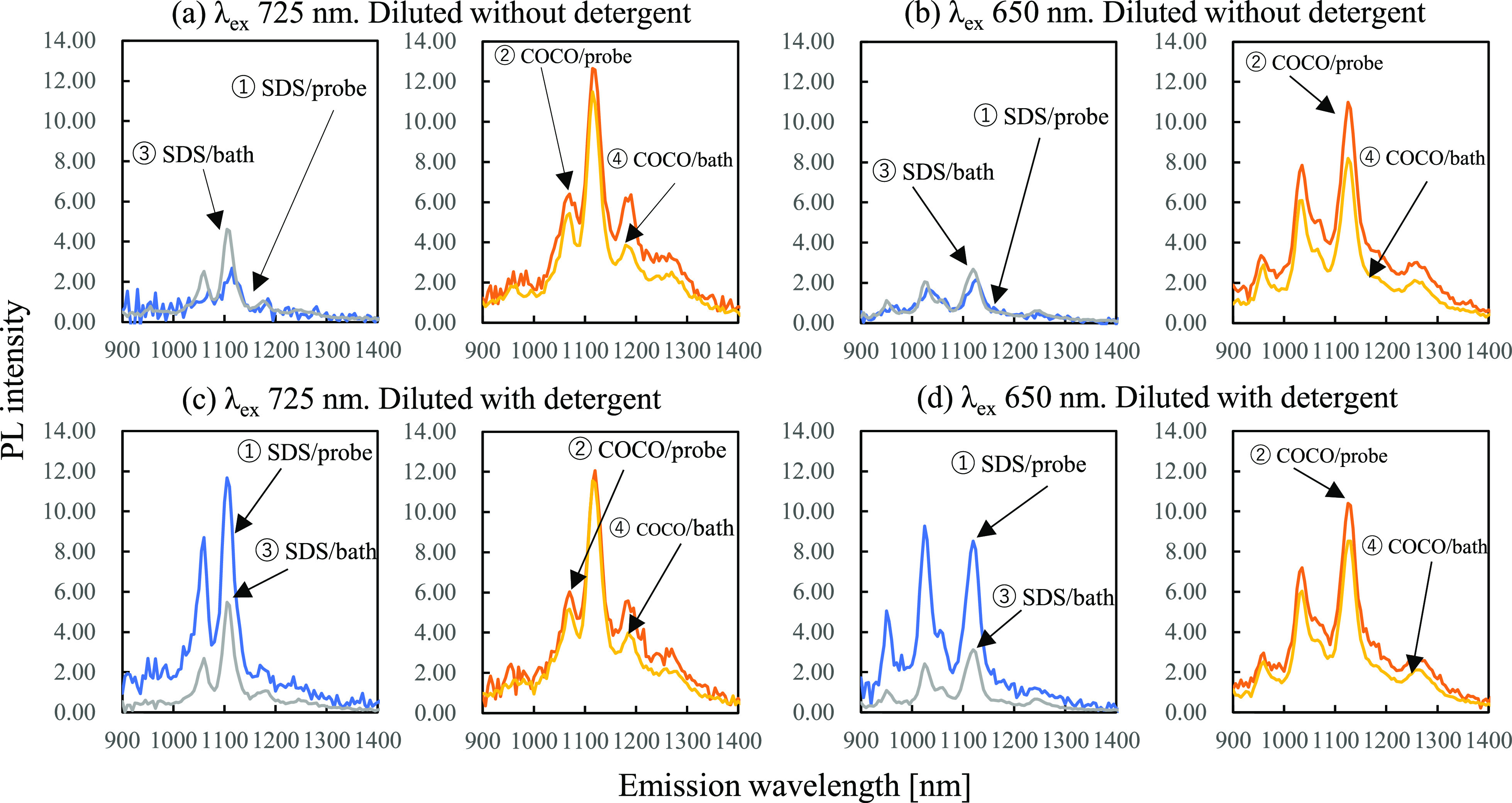
Cross sections of the PL maps of the single-walled CNT suspensions prepared using ① SDS via probe-type sonication, ② COCO via probe-type sonication, ③ SDS via bath-type sonication, and ④ COCO via bath-type sonication at excitation wavelengths of (a,c) 725 and (b,d) 650 nm. Each suspension was diluted with detergent-free [(a,b) or detergent (1% SDS and 1% COCO)-containing phosphate buffer solutions (c,d) respectively], such that the absorbance of the final suspension was 0.1 in order to measure PL within appropriate detection ranges of the spectrometer. Then, as shown in the figure, PL intensity values were converted into those of the original suspensions without dilution.

The UV–vis absorbance spectra of the supernatants of the SWNT suspensions subjected to centrifugation at 1500 rpm for 180 min are presented in Figure 2. The samples were diluted 10 times using the same procedure described for the stability experiments, and the absorbance spectra were measured immediately after dilution. In Figure 2, absorbance values were converted into those of the original suspension for simple comparison with Figures 3 and 4. The raw data of obtained spectra are shown in Figure S2. The spectra of the SDS- and COCO-containing SWNT suspensions were compared to determine the concentration of SWNTs of each sample and not to evaluate the stability of the SWNT suspensions.

The spectra represent the average data from three independent experiments. The SWNT concentrations of the suspensions subjected to probe-type sonication were higher than those of the suspensions subjected to bath-type sonication (Figure 2, lines ① and ②). The dispersion efficiency of the COCO/bath samples was much higher than that of the SDS/bath samples. Therefore, bath-type sonication might be milder than probe-type sonication. This suggested that COCO can effectively disperse SWNTs even under mild sonication conditions. The average absorbances of the SDS/probe, COCO/probe, SDS/bath, and COCO/bath samples at 808 nm were 8.61, 8.39, 1.88, and 3.94, respectively. The dispersion efficiencies of the SDS/probe and COCO/probe samples were 458 and 213% higher than those of the SDS/bath and COCO/bath samples, respectively. Moreover, the dispersion efficiency of the COCO/bath sample was 201% higher than that of SDS/bath sample. The UV–vis absorbances of several SWNT suspensions at different wavelengths are presented in Figure S2 (absorbance of 10 times diluted samples).

The PL maps of the SDS- and COCO-containing SWNT suspensions in the excitation wavelength range of 600–800 nm are illustrated in Figure 3. Because the HiPco-fabricated SWNT powder included SWNTs with several chiralities, several PL spots were observed in the PL maps in the emission wavelength range of 900–1400 nm. SWNT chirality was attributed to the structural differences between SWNTs, and each chirality exhibited different PL wavelengths.

Herein, we focused on the PL spots of the SWNTs with (9, 4) and (7, 6) chiralities (circles in Figure 3). For the PL measurements, all samples were diluted, such that their absorbance at 808 nm was 0.1 in order to measure PL within appropriate detection ranges of the spectrometer. Then, as shown in Figures 3 and 4, measured PL values were converted into those of the original suspensions for simple comparison of each sample.

The top images shown in Figure 3 represent the PL maps of the samples diluted with detergent-free phosphate buffer solution. The detergent concentrations of the diluted SDS/probe, COCO/probe, SDS/bath, and COCO/bath samples were 0.020, 0.015, 0.124, and 0.037%, respectively, which were lower than those of the undiluted samples. During this experiment, the PL signals of the SDS/probe samples (①, Figure 3a) were weak. Conversely, the PL signals of the SDS/bath samples were distinct (③, Figure 3a). The intensities of the PL signals of the COCO/probe and COCO/bath samples were strong (② and ④, respectively, Figure 3a).

The PL spots of the samples diluted with detergent (1% SDS or 1% COCO)-containing phosphate buffer solutions were distinct (①–④, Figure 3b). These results suggested that the SDS-containing SWNT suspensions became unstable upon decreasing the SDS concentration. In fact, distinct aggregates were observed when the SDS-containing SWNT suspensions were diluted with a detergent-free phosphate buffer solution (Figure 1). We hypothesized that small aggregates started to form during the PL measurements, although the PL measurements were performed immediately after the samples were diluted. These results indicated that COCO was effective for preparing stable SWNT suspensions.

The cross sections of the PL maps shown in Figure 3 are illustrated in Figure 4. PL intensity values (*Y* axis) were converted into those of the original suspensions although PL measurements were carried out with the diluted samples. The raw data without conversion are shown in Figure S4. The brightest peak in the PL maps of the SWNT suspensions corresponded to the SWNT with a (9, 4) chirality irradiated with 725 nm light (Figure 4a,c), followed by the peak corresponding to the SWNT with a (7, 6) chirality excited by 650 nm light (Figure 4b,d). The PL intensity of the SDS-containing SWNT suspension decreased significantly upon diluting the suspension with a detergent-free phosphate buffer solution (Figure 4a,b). Conversely, the PL intensities of the SDS- and COCO- containing SWNT suspensions diluted with detergent (1% SDS or 1% COCO)-containing phosphate buffer solutions were similar. The samples subjected to probe- and bath-type sonication presented similar PL intensity trends; moreover, the PL intensity trends of the SWNTs with (9, 4) and (7, 6) chiralities were similar. These results suggested that the SDS-containing SWNT suspension became unstable upon decreasing the SDS concentration, whereas the COCO-containing SWNT suspension remained stable even after dilution.

The PL intensity of monodispersed SWNTs is much stronger than that of bundled SWNTs.^7−11^ The SWNT concentration of the SWNT suspensions prepared via probe-type sonication was higher than that of the SWNT suspensions prepared via bath-type sonication (Figure 2). The SWNT concentration of the suspensions fabricated via bath-type sonication was lower than that of the SWNT suspensions fabricated via probe-type sonication, indicating that the sonication efficiency of the bath-type sonicator was lower than that of the probe-type sonicator. Higher efficiency of the probe-type sonication can be explained that it directly provides strong power to SWNTs at the top of the probe. The bath-type sonication was mild because ultrasonic effects were indirectly provided through a glass vial.

On the other hand, after centrifugation to remove aggregates, it seems that most of the SWNT suspensions fabricated via bath-type sonication were monodispersed, although the SWNT concentrations of the suspensions were low (comparison of Figures 4 and S4). It seems that the ratio of monodispersed SWNTs was higher in suspensions prepared by bath-type sonication after centrifugation. In other words, suspensions prepared via bath-type sonication might include bigger aggregates; then, the aggregates might be well removed by centrifugation. If so, bath-type sonication was effective for preparing SWNT suspensions for PL measurements, although the dispersion efficiency of bath-type sonication was low.

The numerical analysis of the cross sections shown in Figure 4 is presented in Table 1. The measured PL values were converted into those of the original suspensions although actual measurements were carried out with diluted samples. The table with raw data without conversion is indicated in Table S1. The PL intensity of the COCO-dispersed SWNTs was higher than that of the SDS-dispersed SWNTs. In addition, PL peak shifts were observed, especially in the COCO-dispersed SWNT suspensions. Considering the PL wavelengths of the SWNT suspension containing 1% SDS to be the standard wavelengths [1105 and 1120 nm for the SWNTs with (9, 4) and (7, 6) chiralities, respectively], the COCO-dispersed (9, 4) and (7, 6) SWNTs red shifted by 10–15 and 5 nm, respectively. Typically, the dielectric constants of adsorbed molecules affect the PL intensity and wavelengths of SWNTs.^53−57^ We hypothesized that the differences in the physicochemical properties of SDS and COCO induced the differences in peak shifts between the SDS- and COCO-dispersed SWNT suspensions. The PL wavelength of the SDS-dispersed SWNTs changed when the suspension was diluted with SDS-free phosphate buffer solution. This was attributed to the detachment of SDS from the surface of SWNTs upon dilution leading to the formation of SWNT aggregates.

**Table 1:** PL Intensities and Peak Shifts of the Single-Walled CNT Suspensions Prepared Using ① SDS Via Probe-Type Sonication, ②COCO Via Probe-Type Sonication, ③ SDS Via Bath-Type Sonication, and ④ COCO and Bath-Type Sonication^a^

		(9, 4) λ_ex_ 725 nm		(7, 6) λ_ex_ 650 nm	
dispersion	phosphate buffer with	PL of peak	peak shift [nm]	PL of peak	peak shift [nm]
① SDS, probe	0% SDS	2.67 ± 0.60 (1115 nm)	+10	2.15 ± 0.86 (1125 nm)	+5
② COCO, probe	0% COCO	12.67 ± 0.34 (1115 nm)	+10	10.99 ± 0.59 (1125 nm)	+5
③ SDS, bath	0% SDS	4.61 ± 0.15 (1105 nm)	0	2.67 ± 0.15 (1120 nm)	0
④ COCO, bath	0% COCO	11.47 ± 0.71 (1115 nm)	+10	8.20 ± 0.63 (1125 nm)	+5
① SDS, probe	1% SDS	11.71 ± 1.64 (1105 nm)	0	8.52 ± 0.60 (1120 nm)	0
② COCO, probe	1% COCO	12.09 ± 0.59 (1120 nm)	+15	10.41 ± 0.00 (1125 nm)	+5
③ SDS, bath	1% SDS	5.48 ± 0.56 (1105 nm)	0	3.12 ± 0.15 (1120 nm)	0
④ COCO, bath	1% COCO	11.55 ± 1.18 (1115 nm)	+10	8.52 ± 0.87 (1125 nm)	+5

a PL values were converted into those of the original suspensions although actual measurements were carried out with diluted samples.

Although we do not have clear explanation about the reasons of the difference between SDS and COCO, there is a possible explanation. COCO includes two types of natural surfactants (sodium alkyl ether sulfate and alkyl betaine) although detailed information is not provided by the manufacturer. On the other hand, Madni et al. suggested that combination of two different surfactants is effective to improve solubilization efficiency of CNTs.^58^ Because COCO forms mixed anion and amphoteric ions as micelles, the critical micelle concentration of micelles of COCO might be lower than that of SDS. It might be helpful for better solubilization.^58^

The suspensions were evaluated by Raman spectroscopy. All samples showed no significant changes in G/D ratios. We think that SWNT structures were not collapsed by sonications.

**Figure 5:**
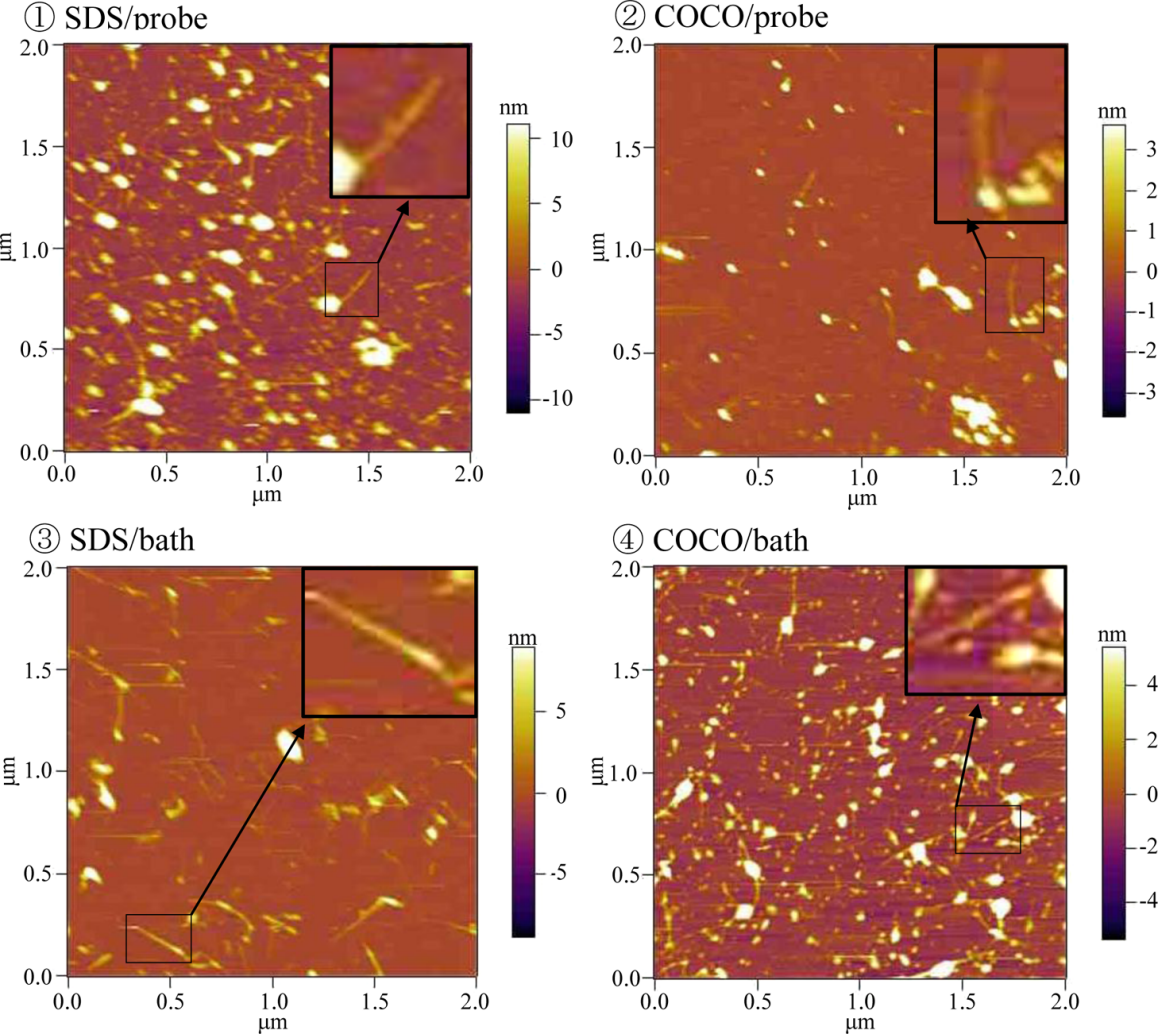
Atomic force microscopy images of single-walled CNT suspensions prepared using ① SDS via probe-type sonication, ②COCO via probe-type sonication, ③ SDS via bath-type sonication, and ④ COCO via bath-type sonication. Each suspension was diluted 100 times with detergent-containing phosphate buffer solutions and analyzed after deposition on the AP-mica surface in aqueous solutions.

Last, we evaluated each suspension using atomic force microscopy (AFM). The AFM images of the SDS/probe, SDS/bath, COCO/probe, and COCO/bath suspensions are presented in Figure 5. Each suspension was diluted with a detergent-free phosphate buffer solution and dropped onto an AP-mica surface. Subsequently, the samples were immediately analyzed using the AFM instrument. Well-dispersed SWNTs were present in all samples. The magnified SWNT images in the insets of Figure 5 ①–④ confirmed that many SWNTs were well monodispersed. AFM measurements are advantageous because they allow for observations in liquids; however, only a few researchers have performed AFM experiments of SWNTs in aqueous solutions.^59−62^ Although we did not quantitatively evaluate the SWNT morphologies in this work, AFM observation in liquids is an attractive research subject to understand interactions between SWNTs and organic molecules based on the fact that adsorption equilibrium is an attractive subject.

## 3 Conclusions

We prepared monodispersed SWNT suspensions using SDS or COCO and probe- or bath-type sonicators. The natural eco-friendly COCO detergent was as effective as SDS for preparing SWNT suspensions. The SWNT suspensions prepared using COCO were more stable than those prepared using SDS. In addition, although the SWNT concentrations of the suspensions prepared via bath-type sonication were lower than those of the suspensions prepared via probe-type sonication, the fraction of monodispersed SWNTs of the suspensions prepared via bath-type sonication was higher than that of the suspensions prepared via probe-type sonication. Our results provide helpful information for developing various biological applications that use the NIR PL of SWNTs.

## 4 Materials and Methods

SWNTs (HS27-122, Raymor Industries Inc., Boisbriand, City, QC, Canada), SDS (192-08672, FUJIFILM Wako Pure Chemical Co., Osaka, Japan), and COCO (4973512322624, Saraya Co. Ltd., Osaka, Japan) were used as received. COCO consisted of 20% sodium alkyl ether sulfate and alkyl betaine detergents.

SWNT powder was mixed with 1 mL of a 20 mM phosphate buffer solution (pH 7.0) containing SDS or COCO. The concentrations of SWNTs and detergents in the final mixture were 0.5 mg/L and 1%, respectively. The mixtures were sonicated using a probe-type (VCX-130, Sonics & Materials, Inc., Newtown, CT, USA) or a bath-type sonicator (W-113A, Honda Electronics Co. Ltd, Aichi, Japan). The probe-type sonicated samples were processed for 90 min at a current density of 0 C, an amplitude of 60%, a frequency of 20 kHz, and a power of 130 W. The bath-type sonicated samples were processed for 90 min at a current density of 0 C, frequency of 45 kHz, and power of 100 W.

To evaluate the stability of the suspensions, 300 μL of each sample was diluted with 2700 μL of a detergent-free phosphate buffer solution in a plastic cuvette. The concentration of SDS or COCO was decreased to 0.1%. The samples were photographed immediately after preparation and then 1 and 7 days later. The remaining samples were centrifuged at a rate of 15 000 rpm and a current density of 8 C for 180 min and, thereafter, 70% of each supernatant was used as the SWNT suspension for the subsequent experiments.

For ultraviolet–visible (UV–vis) optical spectroscopy experiments, the centrifuged SWNT suspensions were diluted 10 times with a detergent-free phosphate buffer solution in a two-sided clear quartz cell cuvette. A UV–vis spectrophotometer (V-630, JASCO, Tokyo, Japan) was used to record the absorbance of the samples in the wavelength range of 400–1100 nm.

A PL spectrometer (NIR–PL System, Shimadzu Co., Ltd., Kyoto, Japan) was used to record the PL spectra of the samples. For the PL measurements, each sample was diluted with detergent-free and detergent-containing phosphate buffer solutions in a quadruple clear quartz cell cuvette until the UV–vis absorbance of each sample at 808 nm was 0.1.^63^ The PL profiles of all samples were obtained in the excitation and emission wavelength ranges of 600–800 and 900–1400 nm, respectively.

Raman spectroscopy was carried out by a microscopic Raman spectrometer (RAMANtouch i VIS-NIR, Nanophoton Corporation, Osaka, Japan). 25 μL of original SWNT dispersions was dropped on a glass coverslip and dried in air. Raman spectra were measured with 532 nm excitation wavelength using a 20× objective lens in air.

AFM experiments were performed using an MFP-3D (Asylum Research, Santa Barbara, CA, USA) instrument in a phosphate buffer solution. A BL-AC40TS-C2 (Olympus Co., Tokyo, Japan) cantilever was used for the AC mode measurements. To prepare the samples for the AFM experiments, SWNT suspensions were diluted with a detergent-free phosphate buffer solution. The diluted suspensions were dropped onto a mica surface that was pretreated with a 0.01% solution of 3-aminopropyltriethoxysilane (AP-mica).^64,65^

Each stability evaluation experiment was repeated five times. Each suspension was prepared independently. The results of the three middle PL spectroscopy measurements for the SWNTs with (9, 4) chirality were used for data analysis. Conversely, the PL intensity and PL peak shift of each suspension were measured three times to eliminate fluctuations in the preparation procedures, such as sonication.

## Supporting Information Available

Absorption spectrum in the original SWNT dispersion; PL map and spectrum in the original SWNT dispersion; and Raman spectrum of SDS-SWNT, COCO-SWNT, and SWNT powder (PDF)

## Abbreviations

AFM: atomic force microscopy
AP-mica: mica treated with 3-aminopropyltriethoxysilane
CNT: carbon nanotube
COCO: coconut-based natural detergent
HiPco: high-pressure carbon monoxide
PL: photoluminescence
NIR: near-infrared
SC: sodium cholate
SDS: sodium dodecyl sulfate
SWNT: single-walled carbon nanotube
UV–vis: ultraviolet–visible

## References

[ref1] Ma P.-C.; Siddiqui N. A.; Marom G.; Kim J.-K. Dispersion and functionalization of carbon nanotubes for polymer-based nanocomposites: A review. Composites, Part A 2010, 41, 1345–1367. 10.1016/j.compositesa.2010.07.003.

[ref2] Moore V. C.; Strano M. S.; Haroz E. H.; Hauge R. H.; Smalley R. E.; Schmidt J.; Talmon Y. Individually suspended single-walled carbon nanotubes in various surfactants. Nano Lett. 2003, 3, 1379–1382. 10.1021/nl034524j.

[ref3] Nakamura G.; Tanaka Y.; Niidome Y.; Nakashima N. Efficient Solubilization of Single-Walled Carbon Nanotubes Using Tea Solutions. J. Nanosci. Nanotechnol. 2010, 10, 3815–3821. 10.1166/jnn.2010.2014. 20355373

[ref4] Vaisman L.; Wagner H. D.; Marom G. The role of surfactants in dispersion of carbon nanotubes. Adv. Colloid Interface Sci. 2006, 128–130, 37–46. 10.1016/j.cis.2006.11.007. 17222381

[ref5] Yan X.; Zhai Z.; Xu J.; Song Z.; Shang S.; Rao X. Hybrids of CO2-Responsive Water-Redispersible Single-Walled Carbon Nanotubes by a Surfactant Based on Natural Rosin. ACS Omega 2019, 4, 19478–19482. 10.1021/acsomega.9b03027. 31763572 PMC6868910

[ref6] Zheng M.; Jagota A.; Semke E. D.; Diner B. A.; McLean R. S.; Lustig S. R.; Richardson R. E.; Tassi N. G. DNA-assisted dispersion and separation of carbon nanotubes. Nat. Mater. 2003, 2, 338–342. 10.1038/nmat877. 12692536

[ref7] Graf A.; Zakharko Y.; Schiessl S. P.; Backes C.; Pfohl M.; Flavel B. S.; Zaumseil J. Large scale, selective dispersion of long single-walled carbon nanotubes with high photoluminescence quantum yield by shear force mixing. Carbon 2016, 105, 593–599. 10.1016/j.carbon.2016.05.002.

[ref8] Ito M.; Kobayashi T.; Ito Y.; Hayashida T.; Nii D.; Umemura K.; Homma Y. Intense photoluminescence from dried double-stranded DNA and single-walled carbon nanotube hybrid. Appl. Phys. Lett. 2014, 104, 043102 10.1063/1.4863272.

[ref9] Minami N.; Kim Y. J.; Miyashita K.; Kazaoui S.; Nalini B. Cellulose derivatives as excellent dispersants for single-wall carbon nanotubes as demonstrated by absorption and photoluminescence spectroscopy. Appl. Phys. Lett. 2006, 88, 093123 10.1063/1.2180870.

[ref10] Okamoto M.; Fujigaya T.; Nakashima N. Individual dissolution of single-walled carbon nanotubes by using polybenzimidazole, and highly effective reinforcement of their composite films. Adv. Funct. Mater. 2008, 18, 1776–1782. 10.1002/adfm.200701257.

[ref11] Stürzl N.; Hennrich F.; Lebedkin S.; Kappes M. M. Near Monochiral Single-Walled Carbon Nanotube Dispersions in Organic Solvents. J. Phys. Chem. C 2009, 113, 14628–14632. 10.1021/jp902788y.

[ref12] Ishibashi Y.; Ito M.; Homma Y.; Umemura K. Monitoring the antioxidant effects of catechin using single-walled carbon nanotubes: Comparative analysis by near-infrared absorption and near-infrared photoluminescence. Colloids Surf., B 2018, 161, 139–146. 10.1016/j.colsurfb.2017.10.055. 29073526

[ref13] Luo Z.; Pfefferle L. D.; Haller G. L.; Papadimitrakopoulos F. (n, m) abundance evaluation of single-walled carbon nanotubes by fluorescence and absorption spectroscopy. J. Am. Chem. Soc. 2006, 128, 15511–15516. 10.1021/ja0657096. 17132018

[ref14] McDonald T. J.; Jones M.; Engtrakul C.; Ellingson R. J.; Rumbles G.; Heben M. J. Near-infrared Fourier transform photoluminescence spectrometer with tunable excitation for the study of single-walled carbon nanotubes. Rev. Sci. Instrum. 2006, 77, 053104 10.1063/1.2198748.

[ref15] Umemura K.; Ishibashi Y.; Ito M.; Homma Y. Quantitative Detection of the Disappearance of the Antioxidant Ability of Catechin by Near-Infrared Absorption and Near-Infrared Photoluminescence Spectra of Single-Walled Carbon Nanotubes. ACS Omega 2019, 4, 7750–7758. 10.1021/acsomega.9b00767. 31459864 PMC6648150

[ref16] Cognet L.; Tsyboulski D. A.; Rocha J.-D. R.; Doyle C. D.; Tour J. M.; Weisman R. B. Stepwise quenching of exciton fluorescence in carbon nanotubes by single-molecule reactions. Science 2007, 316, 1465–1468. 10.1126/science.1141316. 17556581

[ref17] Jeng E. S.; Moll A. E.; Roy A. C.; Gastala J. B.; Strano M. S. Detection of DNA hybridization using the near-infrared band-gap fluorescence of single-walled carbon nanotubes. Nano Lett. 2006, 6, 371–375. 10.1021/nl051829k. 16522025 PMC6438164

[ref18] Liu Z.; Tabakman S. M.; Chen Z.; Dai H. Preparation of carbon nanotube bioconjugates for biomedical applications. Nat. Protoc. 2009, 4, 1372–1381. 10.1038/nprot.2009.146. 19730421 PMC2853228

[ref19] Yamazaki Y.; Umemura K. Sensing of epigallocatechin gallate and tannic acid based on near infrared optical spectroscopy of DNA-wrapped single-walled carbon nanotube hybrids. J. Near Infrared Spectrosc. 2021, 29, 73–83. 10.1177/0967033520982354.

[ref20] Zhang J.; Boghossian A. A.; Barone P. W.; Rwei A.; Kim J.-H.; Lin D.; Heller D. A.; Hilmer A. J.; Nair N.; Reuel N. F.; Strano M. S. Single Molecule Detection of Nitric Oxide Enabled by d(AT)(15) DNA Adsorbed to Near Infrared Fluorescent Single-Walled Carbon Nanotubes. J. Am. Chem. Soc. 2011, 133, 567–581. 10.1021/ja1084942. 21142158

[ref21] Cathcart H.; Quinn S.; Nicolosi V.; Kelly J. M.; Blau W. J.; Coleman J. N. Spontaneous debundling of single-walled carbon nanotubes in DNA-based dispersions. J. Phys. Chem. C 2007, 111, 66–74. 10.1021/jp065503r.

[ref22] Hasan T.; Scardaci V.; Tan P.; Rozhin A. G.; Milne W. I.; Ferrari A. C. Stabilization and ″Debundling″ of single-wall carbon nanotube dispersions in N-Methyl-2-pyrrolidone (NMP) by polyvinylpyrrolidone (PVP). J. Phys. Chem. C 2007, 111, 12594–12602. 10.1021/jp0723012.

[ref23] Koyama T.; Asaka K.; Hikosaka N.; Kishida H.; Saito Y.; Nakamura A. Femtosecond luminescence decay due to exciton energy transfer in single-walled carbon nanotube bundles. J. Lumin. 2011, 131, 494–497. 10.1016/j.jlumin.2010.10.044.

[ref24] Murakami T.; Kisoda K.; Tokuda T.; Matsumoto K.; Harima H.; Mitikami K.; Isshiki T. Raman and photoluminescence from dispersed single walled carbon nanotubes. Diamond Relat. Mater. 2007, 16, 1192–1194. 10.1016/j.diamond.2007.01.012.

[ref25] Almanassra I. W.; Manasrah A. D.; Al-Mubaiyedh U. A.; Al-Ansari T.; Malaibari Z. O.; Atieh M. A. An experimental study on stability and thermal conductivity of water/CNTs nanofluids using different surfactants: A comparison study. J. Mol. Liq. 2020, 304, 111025 10.1016/j.molliq.2019.111025.

[ref26] Duan W. H.; Wang Q.; Collins F. Dispersion of carbon nanotubes with SDS surfactants: a study from a binding energy perspective. Chem. Sci. 2011, 2, 1407–1413. 10.1039/c0sc00616e.

[ref27] Halelfadl S.; Maré T.; Estellé P. Efficiency of carbon nanotubes water based nanofluids as coolants. Exp. Therm. Fluid Sci. 2014, 53, 104–110. 10.1016/j.expthermflusci.2013.11.010.

[ref28] Huang Y. Y.; Terentjev E. M. Dispersion of Carbon Nanotubes: Mixing, Sonication, Stabilization, and Composite Properties. Polymers 2012, 4, 275–295. 10.3390/polym4010275.

[ref29] Movia D.; Del Canto E.; Giordani S. Spectroscopy of single-walled carbon nanotubes in aqueous surfactant dispersion. Phys. Status Solidi B 2009, 246, 2704–2707. 10.1002/pssb.200982277.

[ref30] Shin J.-Y.; Premkumar T.; Geckeler K. E. Dispersion of single-walled carbon nanotubes by using surfactants: Are the type and concentration important?. Chem.—Eur. J. 2008, 14, 6044–6048. 10.1002/chem.200800357. 18491336

[ref31] Barisci J. N.; Tahhan M.; Wallace G. G.; Badaire S.; Vaugien T.; Maugey M.; Poulin P. Properties of carbon nanotube fibers spun from DNA-stabilized dispersions. Adv. Funct. Mater. 2004, 14, 133–138. 10.1002/adfm.200304500.

[ref32] Chou S. G.; Ribeiro H. B.; Barros E. B.; Santos A. P.; Nezich D.; Samsonidze G. G.; Fantini C.; Pimenta M. A.; Jorio A.; Plentz F.; Dresselhaus M. S.; Dresselhaus G.; Saito R.; Zheng M.; Onoa G. B.; Semke E. D.; Swan A. K.; Unlu M. S.; Goldberg B. B. Optical characterization of DNA-wrapped carbon nanotube hybrids. Chem. Phys. Lett. 2004, 397, 296–301. 10.1016/j.cplett.2004.08.117.

[ref33] Liu P. Modifications of carbon nanotubes with polymers. Eur. Polym. J. 2005, 41, 2693–2703. 10.1016/j.eurpolymj.2005.05.017.

[ref34] Wang D.; Chen L. Temperature and pH-responsive single-walled carbon nanotube dispersions. Nano Lett. 2007, 7, 1480–1484. 10.1021/nl070172v. 17488048

[ref35] Xu Y.; Pehrsson P. E.; Chen L.; Zhang R.; Zhao W. Double-stranded DNA single-walled carbon nanotube hybrids for optical hydrogen peroxide and glucose sensing. J. Phys. Chem. C 2007, 111, 8638–8643. 10.1021/jp0709611.

[ref36] Du M.; Yang T.; Jiao K. Immobilization-free direct electrochemical detection for DNA specific sequences based on electrochemically converted gold nanoparticles/graphene composite film. J. Mater. Chem. 2010, 20, 9253–9260. 10.1039/c0jm01549k.

[ref37] Strano M. S.; Zheng M.; Jagota A.; Onoa G. B.; Heller D. A.; Barone P. W.; Usrey M. L. Understanding the nature of the DNA-assisted separation of single-walled carbon nanotubes using fluorescence and Raman spectroscopy. Nano Lett. 2004, 4, 543–550. 10.1021/nl034937k.

[ref38] Tu X.; Manohar S.; Jagota A.; Zheng M. DNA sequence motifs for structure-specific recognition and separation of carbon nanotubes. Nature 2009, 460, 250–253. 10.1038/nature08116. 19587767

[ref39] Periyasamy C.; Manickam C. Experimental studies on stability of multi walled carbon nanotube with different oil based nanofluids. Therm. Sci. 2020, 24, 533–539. 10.2298/tsci190412432p.

[ref40] Jiju G.; Wang J.; Ding Y. P.; Lv T.; Xu X. F. Dispersion stability and enhanced heat transfer of cutting use nanofluids prepared by composite of carbon nanotubes and Dialkyl pentasulfide. Mater. Res. Express 2019, 6, 085633 10.1088/2053-1591/ab555c.

[ref41] Jin C.; Wu Q.; Yang G.; Zhang H.; Zhong Y. Investigation on hybrid nanofluids based on carbon nanotubes filled with metal nanoparticles: Stability, thermal conductivity, and viscosity. Powder Technol. 2021, 389, 1–10. 10.1016/j.powtec.2021.05.007.

[ref42] Rastogi R.; Kaushal R.; Tripathi S. K.; Sharma A. L.; Kaur I.; Bharadwaj L. M. Comparative study of carbon nanotube dispersion using surfactants. J. Colloid Interface Sci. 2008, 328, 421–428. 10.1016/j.jcis.2008.09.015. 18848704

[ref43] Vlasov A. Y.; Venediktova A. V.; Ivanov P. V.; Nikolaeva A. L.; Anufrikov Y. A.; Venediktov V. Y. Aggregative Characteristics of Nanocarbon and of a Stabilizing Surfactant in the Aqueous-Polymer Matrix versus Optical Power Limiting Performance. Phys. Status Solidi B 2019, 256, 1900320 10.1002/pssb.201900320.

[ref44] Bouchard D.; Zhang W.; Powell T.; Rattanaudompol U.-s. Aggregation Kinetics and Transport of Single-Walled Carbon Nanotubes at Low Surfactant Concentrations. Environ. Sci. Technol. 2012, 46, 4458–4465. 10.1021/es204618v. 22443301

[ref45] Hirano A.; Gao W. L.; He X. W.; Kono J. Destabilization of Surfactant-Dispersed Carbon Nanotubes by Anions. Nanoscale Res. Lett. 2017, 12, 81 10.1186/s11671-017-1850-1. 28138897 PMC5280815

[ref46] Nasiri A.; Shariaty-Niasar M.; Rashidi A.; Amrollahi A.; Khodafarin R. Effect of dispersion method on thermal conductivity and stability of nanofluid. Exp. Therm. Fluid Sci. 2011, 35, 717–723. 10.1016/j.expthermflusci.2011.01.006.

[ref47] Silvera-Batista C. A.; Weinberg P.; Butler J. E.; Ziegler K. J. Long-Term Improvements to Photoluminescence and Dispersion Stability by Flowing SDS-SWNT Suspensions through Microfluidic Channels. J. Am. Chem. Soc. 2009, 131, 12721–12728. 10.1021/ja903705k. 19678679

[ref48] Singh K.; Sharma S. K.; Gupta S. M. Preparation of Long Duration Stable CNT Nanofluid Using SDS. Integr. Ferroelectr. 2020, 204, 11–22. 10.1080/10584587.2019.1674981.

[ref49] Zhu M.; Peng J.; Tang P.; Qiu F. Preparation and Characterization of Highly Stable and Aqueous Dispersion of Conjugated Polyelectrolyte/Single-Walled Carbon Nanotube Nanocomposites. Acta Chim. Sin. 2018, 76, 453–459. 10.6023/a18030090.

[ref50] Umemura K.; Hamano R.; Komatsu H.; Ikuno T.; Siswoyo E. Dispersion of Carbon Nanotubes with ″Green″ Detergents. Molecules 2021, 26, 2908 10.3390/molecules26102908. 34068851 PMC8153609

[ref51] Umemura K.; Sato Y.; Ishibashi Y.; Ito M.; Homma Y. Various responses of single-walled carbon nanotubes with differing chirality: A suggestion for biosensing. J. Near Infrared Spectrosc. 2020, 28, 51–56. 10.1177/0967033519891666.

[ref52] Vora P. M.; Tu X.; Mele E. J.; Zheng M.; Kikkawa J. M. Chirality dependence of the K-momentum dark excitons in carbon nanotubes. Phys. Rev. B: Condens. Matter Mater. Phys. 2010, 81, 155123 10.1103/physrevb.81.155123.

[ref53] He X.; Velizhanin K. A.; Bullard G.; Bai Y.; Olivier J.-H.; Hartmann N. F.; Gifford B. J.; Kilina S.; Tretiak S.; Htoon H.; Therien M. J.; Doorn S. K. Solvent- and Wavelength-Dependent Photoluminescence Relaxation Dynamics of Carbon Nanotube sp(3) Defect States. ACS Nano 2018, 12, 8060–8070. 10.1021/acsnano.8b02909. 29995379

[ref54] Noé J. C.; Nutz M.; Reschauer J.; Morell N.; Tsioutsios I.; Reserbat-Plantey A.; Watanabe K.; Taniguchi T.; Bachtold A.; Högele A. Environmental Electrometry with Luminescent Carbon Nanotubes. Nano Lett. 2018, 18, 4136–4140. 10.1021/acs.nanolett.8b00871. 29921119 PMC6692058

[ref55] Ohno Y.; Iwasaki S.; Murakami Y.; Kishimoto S.; Maruyama S.; Mizutani T. Excitonic transition energies in single-walled carbon nanotubes: Dependence on environmental dielectric constant. Phys. Status Solidi B 2007, 244, 4002–4005. 10.1002/pssb.200776124.

[ref56] Park J.; Reid O. G.; Blackburn J. L.; Rumbles G. Photoinduced spontaneous free-carrier generation in semiconducting single-walled carbon nanotubes. Nat. Commun. 2015, 6, 8809 10.1038/ncomms9809. 26531728 PMC4667683

[ref57] Uryu S.; Ando T. Environment effect on cross-polarized excitons in carbon nanotubes. Phys. Rev. B: Condens. Matter Mater. Phys. 2012, 86, 125412 10.1103/physrevb.86.125412.

[ref58] Madni I.; Hwang C. Y.; Park S. D.; Choa Y. H.; Kim H. T. Mixed surfactant system for stable suspension of multiwalled carbon nanotubes. Colloids Surf., A 2010, 358, 101–107. 10.1016/j.colsurfa.2010.01.030.

[ref59] Hayashida T.; Umemura K. Atomic Force Microscopy of DNA-wrapped Single-walled Carbon Nanotubes in Aqueous Solution. Colloids Surf., B 2016, 143, 526–531. 10.1016/j.colsurfb.2016.03.068. 27045980

[ref60] Umemura K.; Izumi K.; Oura S. Probe Microscopic Studies of DNA Molecules on Carbon Nanotubes. Nanomaterials 2016, 6, 180 10.3390/nano6100180. PMC5245195 28335308

[ref61] Umemura K.; Sato S. Scanning Techniques for Nanobioconjugates of Carbon Nanotubes. Scanning 2018, 2018, 6254692 10.1155/2018/6254692. 30008981 PMC6020491

[ref62] Umemura K.; Ishizaka K.; Nii D.; Izumi K. Non-uniform binding of single-stranded DNA binding proteins to hybrids of single-stranded DNA and single-walled carbon nanotubes observed by atomic force microscopy in air and in liquid. Appl. Surf. Sci. 2016, 388, 381–384. 10.1016/j.apsusc.2015.12.144.

[ref63] Tomura A.; Umemura K. A convenient method of attaching fluorescent dyes on single-walled carbon nanotubes pre-wrapped with DNA molecules. Anal. Biochem. 2018, 547, 1–6. 10.1016/j.ab.2018.02.004. 29428378

[ref64] Hayashida T.; Umemura K. Surface morphology of hybrids of double-stranded DNA and single-walled carbon nanotubes studied by atomic force microscopy. Colloids Surf., B 2013, 101, 49–54. 10.1016/j.colsurfb.2012.06.018. 22796771

[ref65] Hirayama S.; Hayashida T.; Umemura K. Atomic force microscopy imaging of dialyzed single-walled carbon nanotubes dispersed with sodium dodecyl sulfate. Int. J. Smart Nano Mater. 2013, 4, 119–127. 10.1080/19475411.2012.742170.

