# Supplementary material for: Stable Near-Infrared Photoluminescence of Single-Walled Carbon Nanotubes Dispersed Using a Coconut-Based Natural Detergent
Source: ACS Omega. 2021 Nov 2;6(45):30708–15. doi: 10.1021/acsomega.1c04615 (PMC8603184; doi:10.1021/acsomega.1c04615)
Supplement: Supplementary file 1 — ao1c04615_si_001.pdf [file ao1c04615_si_001.pdf]

Supporting Information

Stable Near-Infrared Photoluminescence of Single-Walled Carbon Nanotubes Dispersed Using a Coconut-Based Natural Detergent

Kota Hirayama, Masaki Kitamura, Ryo Hamano, Kazuo Umemura*

Department of Physics, Tokyo University of Science, 1-3 Kagurazaka, Shinjuku, 1628601, Japan

*Corresponding Author meicun2006@163.com

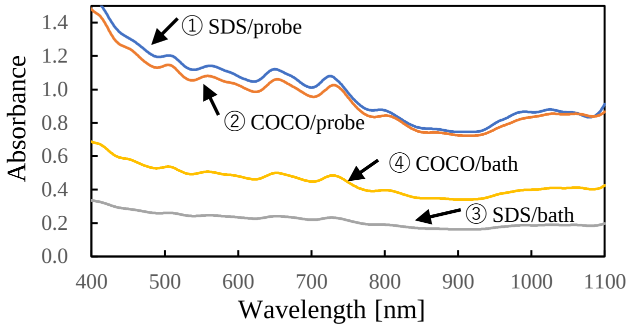

Figure S1 Absorbance spectra of single-walled carbon nanotube suspensions prepared using ① sodium dodecyl sulfate (SDS) via probe-type sonication, ② the coconut-based natural detergent (COCO) via probe-type sonication, ③ SDS via bath-type sonication, and ④ COCO via bath-type sonication. For the absorbance measurements, suspensions were diluted 10 times with a detergent-free phosphate buffer solution until the detergent concentration was 0.1%, and the absorbance of each sample was measured immediately after dilution.

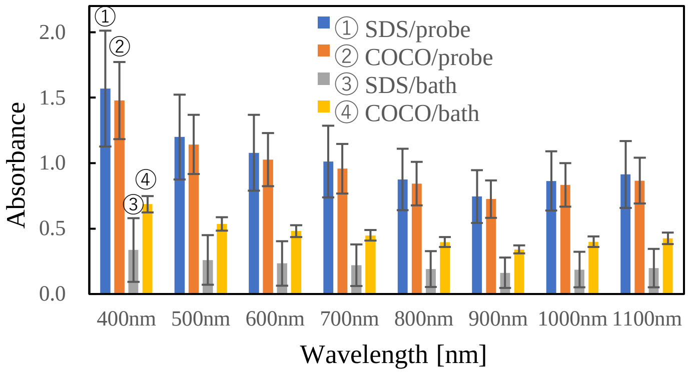

Figure S2 Absorbance of dispersed SWNTs at 400, 500, 600, 700, 800, 900, 1000, and 1100 nm.

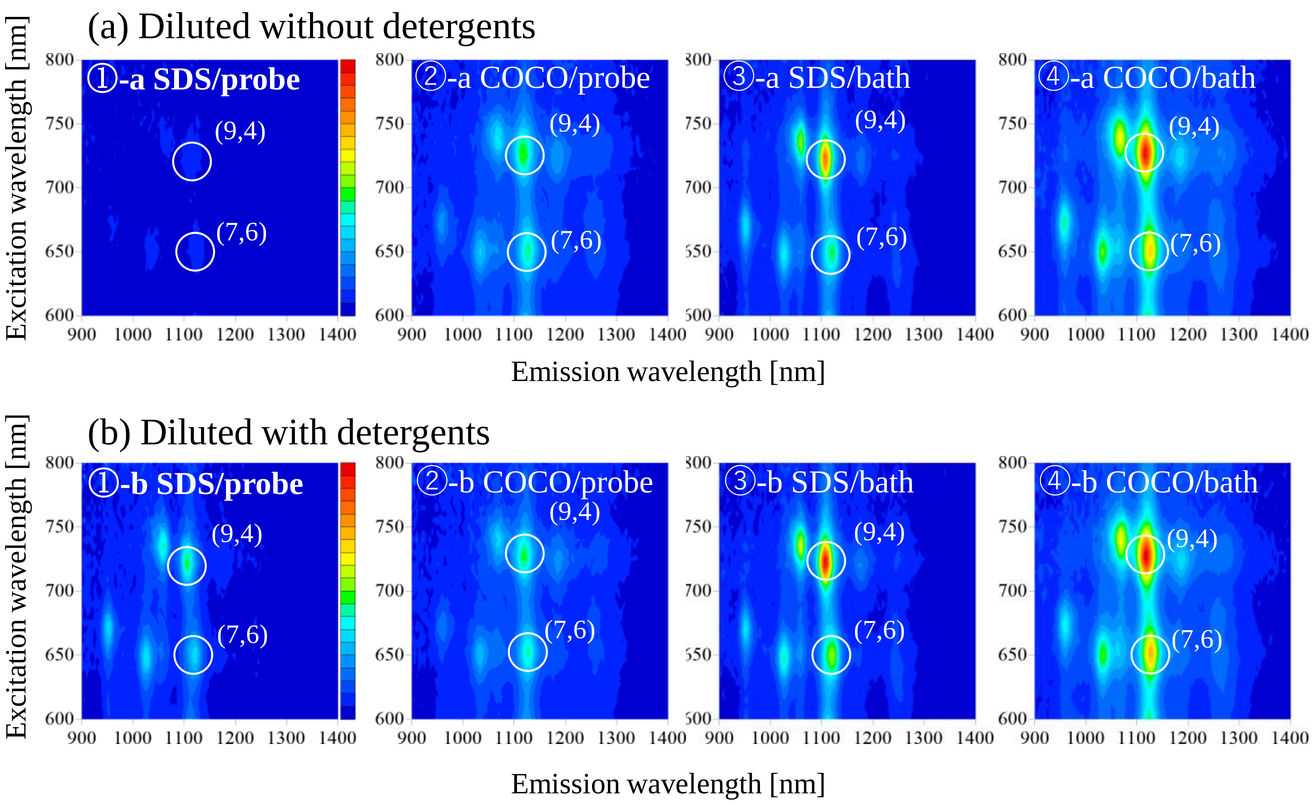

Figure S3 Photoluminescence maps of single-walled carbon nanotube suspensions prepared using ① sodium dodecyl sulfate (SDS) via probe-type sonication, ② the coconut-based natural detergent (COCO) via probe-type sonication, ③ SDS via bath-type sonication, and ④ COCO via bath-type sonication. The excitation and emission wavelengths ranges were 600–800 nm and 900–1400 nm, respectively. Each suspension was diluted with (a) a detergent-free, or (b) a detergent (1% SDS or 1% COCO)-containing phosphate buffer solution, such that absorbance of the suspension was 0.1.

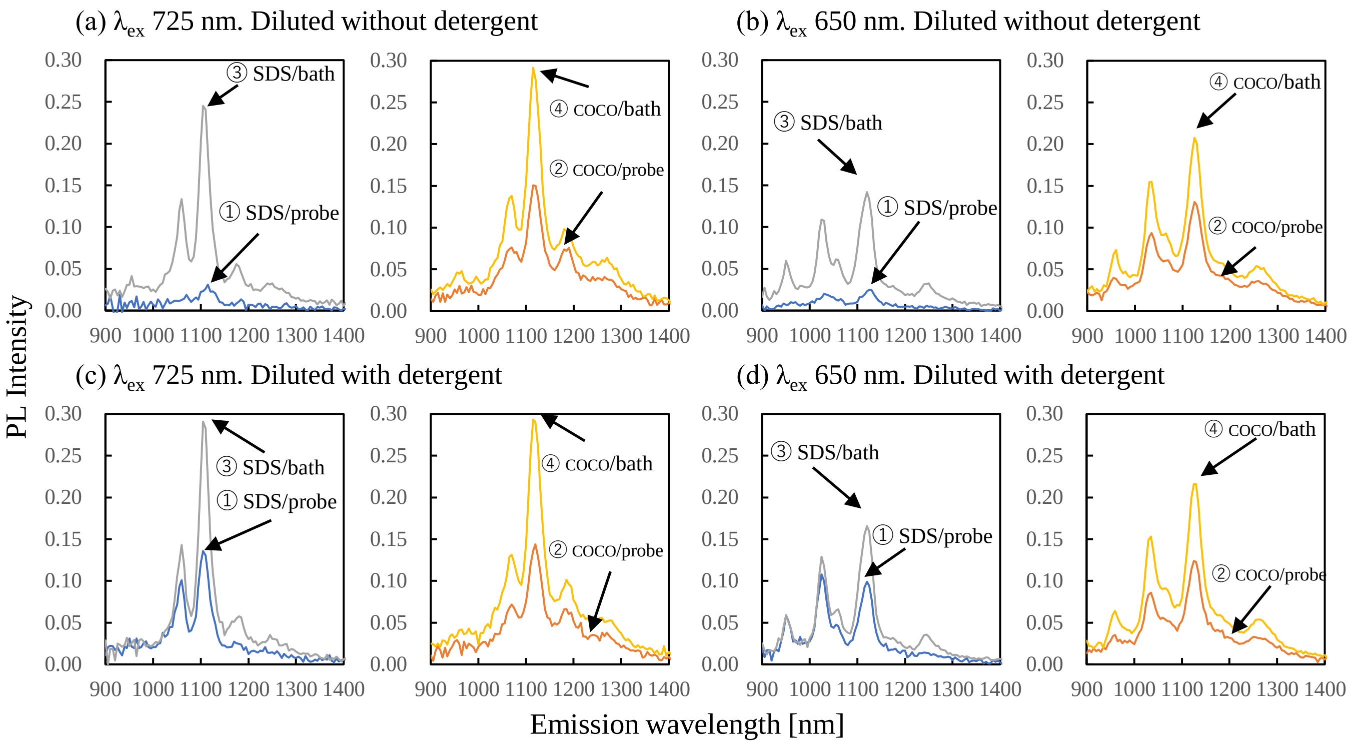

Figure S4 Cross sections of the photoluminescence maps of the single-walled carbon nanotube suspensions prepared using ① sodium dodecyl sulfate (SDS) via probe-type sonication, ② the coconut-based natural detergent (COCO) via probe-type sonication, ③ SDS via bath-type sonication, and ④ COCO via bath-type sonication at excitation wavelengths of (a) and (c) 725 and (b) and (d) 650 nm. Each suspension was diluted with detergent-free ((a) and (c)) or detergent (1% SDS and 1% COCO)-containing phosphate buffer solutions ((b) and (d), respectively) such that the absorbance of the final suspension was 0.1

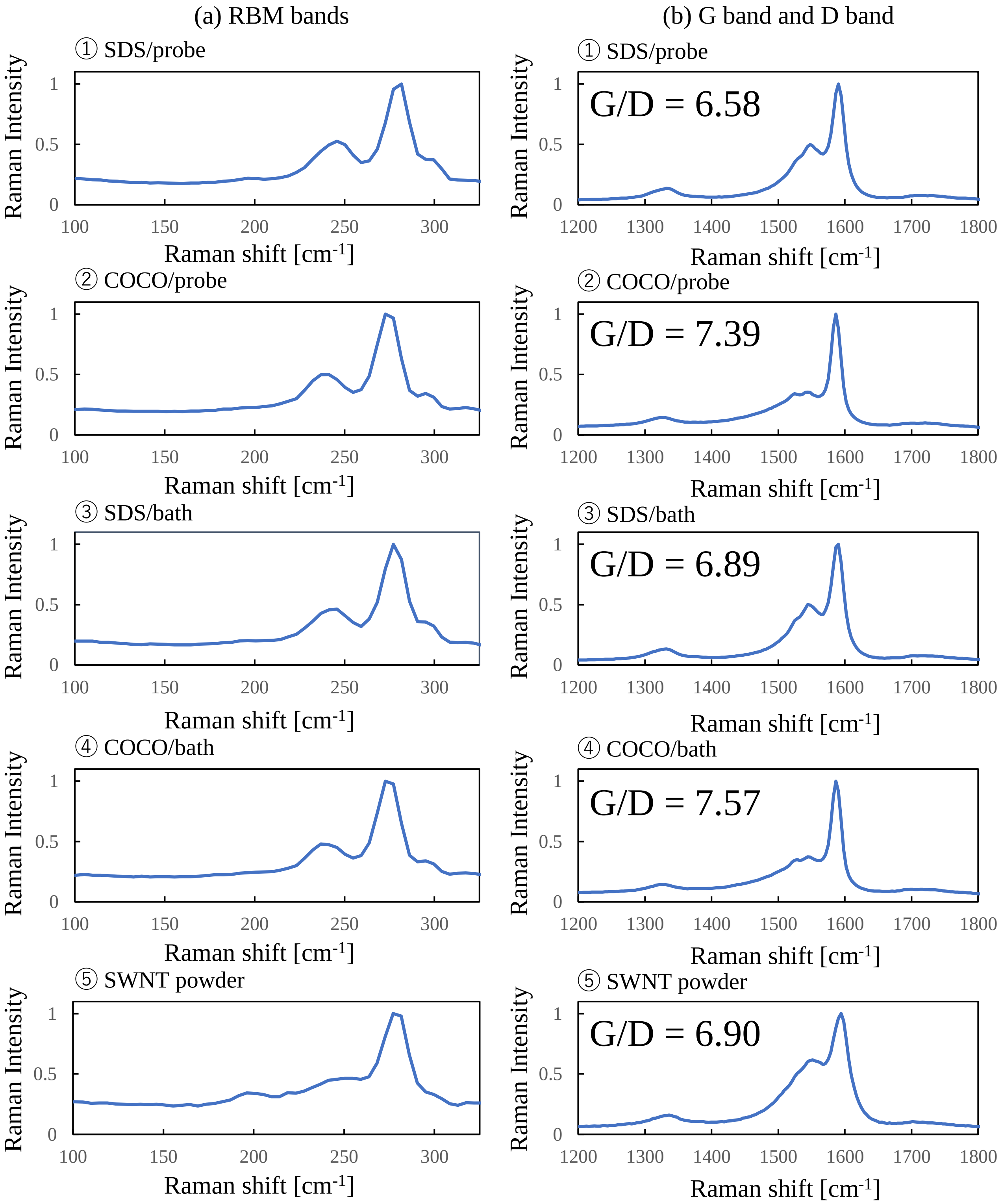

Figure S5 Raman spectra of the single-walled carbon nanotube suspensions. (a) RBM bands. (b) G band and D band. ① sodium dodecyl sulfate (SDS) via probe-type sonication, ② the coconut-based natural detergent (COCO) via probe-type sonication, ③ SDS via bath-type sonication, ④ COCO via bath-type sonication, ⑤ SWNT powder.

		(9,4) λ_{ex} 725nm		(7,6) λ_{ex} 650nm	
Dispersion	Phosphate buffer with	PL of peak	Peak shfit [nm]	PL of peak	Peak shfit [nm]
① SDS, probe	0% SDS	0.031 ± 0.007 (1115nm)	+10	0.025 ± 0.010 (1125nm)	+5
② COCO, probe	0% COCO	0.151 ± 0.004 (1115nm)	+10	0.131 ± 0.007 (1125nm)	+5
③ SDS, bath	0% SDS	0.245 ± 0.008 (1105nm)	0	0.142 ± 0.008 (1120nm)	0
④ COCO, bath	0% COCO	0.291 ± 0.018 (1115nm)	+10	0.208 ± 0.016 (1125nm)	+5
① SDS, probe	1% SDS	0.136 ± 0.019 (1105nm)	0	0.099 ± 0.007 (1120nm)	0
② COCO, probe	1% COCO	0.144 ± 0.007 (1120nm)	+15	0.124 ± 0.000 (1125nm)	+5
③ SDS, bath	1% SDS	0.291 ± 0.030 (1105nm)	0	0.166 ± 0.008 (1120nm)	0
④ COCO, bath	1% COCO	0.293 ± 0.030 (1115nm)	+10	0.216 ± 0.022 (1125nm)	+5

Table S1 Photoluminescence intensities and peak shifts of the single-walled carbon nanotube suspensions prepared using ① sodium dodecyl sulfate (SDS) via probe-type sonication, ② the coconut-based natural detergent (COCO) via probe-type sonication, ③ SDS via bath-type sonication, and ④ COCO and bath-type sonication.
